# Quantitative c-erbB-2 but not c-erbB-1 mRNA expression is a promising marker to predict minor histopathologic response to neoadjuvant radiochemotherapy in oesophageal cancer

**DOI:** 10.1038/sj.bjc.6601976

**Published:** 2004-06-22

**Authors:** F Miyazono, R Metzger, U Warnecke-Eberz, S E Baldus, J Brabender, E Bollschweiler, W Doerfler, R P Mueller, H P Dienes, T Aikou, A H Hoelscher, P M Schneider

**Affiliations:** 1 Department of Visceral and Vascular Surgery, University of Cologne, Joseph-Stelzmann-Strasse 9, 50931 Cologne, Germany; 2 Institute of Pathology, University of Cologne, Joseph-Stelzmann-Strasse 9, 50931 Cologne, Germany; 3 Institute of Genetics, University of Cologne, Joseph-Stelzmann-Strasse 9, 50931 Cologne, Germany; 4 Department of Radiation Oncology, University of Cologne, Joseph-Stelzmann-Strasse 9, 50931 Cologne, Germany; 5 First Department of Surgery, Kagoshima University School of Medicine, Kagoshima, 8-35-1 Sakuragaoka, 890-8520 Kagoshima, Japan

**Keywords:** growth factor receptor, gene expression, response prediction, radiosensitivity, chemosensitivity, multimodality treatment

## Abstract

We examined the potential of quantitative epidermal growth factor receptor (EGFR, synonym: c-erbB-1) and c-erbB-2 (synonym: HER2/neu) mRNA expression to predict minor or major histopathologic response to neoadjuvant radiochemotherapy (*cis*-platinum, 5-FU, 36 Gy), followed by radical surgical resection, in patients with oesophageal cancer. Tissue samples were collected by endoscopic biopsy prior to treatment. RNA was isolated from biopsies and quantitative real-time reverse transcriptase–polymerase chain reaction assays were performed to determine c-erbB-1 and c-erbB-2 mRNA expression. Relative expression (tumour/paired normal tissue ratio standardised for *β*-actin) was calculated for EGFR and c-erbB-2 mRNA. Expression levels were correlated with the objective histopathologic response in resected specimens. Histomorphologic regression was defined as major response when resected specimens contained less than 10% of residual vital tumour cells, or in case a pathologically complete response was achieved. Expression of c-erbB-1 mRNA was not associated with the degree of histomorphological response. In contrast, the relative expression levels of c-erbB-2 mRNA >1 were not associated with major histopathologic responses (sensitivity 41.6%, specificity 100%), and 10 out of 36 (28%) patients could be unequivocally identified, whose tumours did not respond well to the delivered neoadjuvant radiochemotherapy (*P*<0.01). Quantitative expression levels of c-erbB-2, but not c-erbB-1 mRNA, in pretreatment biopsies appear to predict minor histopathologic response to our neoadjuvant radiochemotherapy protocol. This test could be used to prevent expensive, noneffective and potentially harmful therapies in approximately one-fourth of our patients, and leads to a more individualised type of combined modality treatment.

Patients with locally advanced oesophageal cancers have a poor prognosis when treated exclusively by surgical resection. Therefore, many investigators apply neoadjuvant treatment strategies in an effort to improve survival (Sherman *et al*, 2002). Results from phase III randomised trials are encouraging; however, they revealed that only patients with major histopathologic response will eventually benefit from treatment (Walsh *et al*, 1996; Urba *et al*, 2001; MRC Esophageal Study Group, 2002). In addition, these therapies are expensive and associated with increased therapy-induced complication rates (Kelsen, 2000). Predictive molecular markers indicating response or nonresponse to neoadjuvant treatment would be extremely helpful in selecting patients for future treatment protocols.

Epidermal growth factor receptor (EGFR, synonym: c-erbB-1) and c-erbB-2 (synonym: HER2/neu) are members of the type I growth factor receptor gene family (Gullick, 2001). The c-erbB-1 gene encodes a 170 kDa membrane protein. Under physiologic conditions, binding of epidermal growth factor (EGF) or transforming growth factor-*α* (TGF-*α*) leads to receptor kinase activity, and subsequently a complex cascade of events that can induce cellular proliferation (Harris *et al*, 2003; Jorissen *et al*, 2003). The c-erbB-2 gene encodes a 185 kDa transmembrane glycoprotein (p185) with tyrosine kinase activity (Wang and Hung, 2001).

Overexpression of c-erbB-1 or c-erbB-2 mRNA and/or protein has been implicated in the pathogenesis of various solid tumours including breast, ovarian, lung and oesophageal cancers; however, association with prognosis varied substantially between studies and tumour entities (Meden *et al*, 1994; Singleton *et al*, 1994; Schneider *et al*, 2000; Brabender *et al*, 2001; Esteva *et al*, 2002; Lau *et al*, 2002).

In tumour cell lines, the sensitivity to several chemotherapeutic agents (Kroning *et al*, 1995; Dixit *et al*, 1997; Nouri *et al*, 2000) and radiotherapy (Kwok and Sutherland, 1989; Bonner *et al*, 2002) was significantly modified by EGF or EGFR expression levels. In addition, there is evidence that blocking EGFR could further increase chemo- and radiosensitivity of tumour cells expressing high receptor levels *in vitro* and *in vivo* (Anderson and Jankowski, 2003; Gee and Nicholson, 2003).

Several studies demonstrated an association between the expression of c-erbB-2 and the response to adjuvant chemotherapy in breast cancer (Allred *et al*, 1992; Muss *et al*, 1994; Miles *et al*, 1999; Menard *et al*, 2001). These results, however, are challenged by negative data in other adjuvant or neoadjuvant trials (Rozan *et al*, 1998; Wang *et al*, 2002; Zhang *et al*, 2003). Trial results might also depend on the chemotherapeutic agents used (Muss *et al*, 1994; Wang *et al*, 2002). In addition, anti-p185-specific antibodies enhanced *cis*-platin (CDDP) sensitivity (Hancock *et al*, 1991; Pietras *et al*, 1994) and radiosensitivity (Pietras *et al*, 1999) *in vitro*, and promising results have already been reported in a clinical phase II trial using a combination of recombinant anti-p185-HER2/neu monoclonal antibody plus CDDP in refractory metastatic breast cancer (Pegram *et al*, 1998).

The purpose of this prospective study was to investigate the potential of quantitative c-erbB-1 and c-erbB-2 mRNA expression in pretreatment biopsies to predict a minor or major histopathologic response to neoadjuvant therapy with CDDP, 5-fluorouracil (5-FU) and simultaneous radiation (36 Gy), followed by surgical resection in oesophageal cancers.

## PATIENTS AND METHODS

### Study population, demographic data and neoadjuvant therapy

All patients were recruited from an ongoing clinical trial on neoadjuvant radiochemotherapy for oesophageal cancer. None of the patients had prior radio- and/or chemotherapy.

In all, 36 consecutive patients (median age: 59.6 years, range 29.5–72.7; gender: 29 men, seven women) with locally advanced, resectable oesophageal cancers (cT2-4, Nx, M0, UICC/AJCC TNM Classification) in good general health condition (ECOG performance status 0–1) and normal to moderate risk factors for oesophageal surgery (Bollschweiler *et al*, 2000) were offered standardised neoadjuvant radiochemotherapy. Clinical staging was based on barium swallow, endoscopic ultrasound and CT of the chest and abdomen. Diagnostic laparoscopy was performed in all patients with adenocarcinomas to exclude peritoneal carcinomatosis. CDDP (20 mg m^2^ day^−1^) was administered as short-term infusion on days 1–5 and 5-fluorouracil (1000 mg m^2^ day^−1^) as continuous infusion over 24 h on days 1–5. Radiation therapy was administered by linear accelerators with 10–15 MV photons. Radiation therapy was simulated to encompass the tumour volume with 5 cm cephalo-caudad-margins and 2 cm radial margins, and treatment ports were designed to include enlarged regional nodes based on CT evaluation and endoscopic ultrasound. Radiation was delivered in daily fractions of 1.8 Gy (days 1–5, 8–12, 15–19 and 22–26) to a total dose of 36 Gy using a multiple-field technique. Surgical resection was performed 4–5 weeks following completion of chemoradiation after clinical restaging using the same procedures as for staging, except for laparoscopy. Standardised transthoracic *en bloc* oesophagectomy with two-field lymphadenectomy and reconstruction by gastric tube interposition with either left cervical or high intrathoracic anastomosis was performed in all patients (Schroeder *et al*, 2002). Clinical data of the patients are summarised in Table 1

**Table 1:** Clinical and histopathologic parameters (patients with tumour resection: 36)

**Parameter**	**Subtype**	**Number**	**%**
Histology	Squamous	23	63.9
	Adeno	13	36.1
ypT-category^a^	T0	2	5.6
	T1	1	2.8
	T2	7	19.4
	T3	25	69.4
	T4	1	2.8
ypN-category^b^	N0	15	41.7
	N1	21	58.3
R-category^c^	R0	31	86.1
	R1	5	13.9
	R2	0	0
Grading	G1	0	0
	G2	12	33.3
	G3	24	66.7
Regression grade^d^	I	15	41.7
	II	9	25
	III	10	27.8
	IV	2	5.6

a Histopathologic tumour category after neoadjuvant therapy according to UICC (Union Internationale Contre Le Cancer, 5th edition, 1997).

b Histopathologic lymph node category after neoadjuvant therapy according to UICC (Union Internationale Contre Le Cancer, 5th edition, 1997).

c Residual tumour category according to UICC (Union Internationale Contre Le Cancer, 5th edition, 1997).

d Grade I: >50% vital residual tumour cells (VRTC), grade II: 10–50% VRTC, grade III: nearly complete response (NCR) with <10% VRTC and grade IV: complete response (pCR, ypT0).

.

Informed consent was obtained from each patient and the scientific protocol was approved by the local ethic committee.

### Histomorphologic grading of tumour regression

Since clinical response evaluation after neoadjuvant therapy for oesophageal cancer was known to be highly inaccurate (Adelstein *et al*, 1997), objective histopathologic response analysis was applied using morphologic criteria described by Junker *et al* (1997).

The resected specimens were fixed in 10% formaldehyde, *en bloc* embedded in paraffin and sectioned into 5-*μ*m slices which were stained with haematoxylin and eosin. These sections were used for both histopathologic staging according to the TNM classification system (UICC 5th edition, 1997) and histomorphologic evaluation of the effect of radiochemotherapy. The degree of histomorphologic regression was classified into four categories: grade I: >50% vital residual tumour cells (VRTC), grade II: 10–50% VRTC, grade III: nearly complete response (NCR) with <10% VRTC and grade IV: complete response (pCR, ypT0). This analysis was performed by two independent staff pathologists who were blinded for all other clinical data (SEB and HPD), and there was no interobserver variation in response classification.

Regression grades III and IV were considered as major histomorphologic response (MaHR) compared to grades I and II constituting minor histopathologic response (MiHR).

### Tissue acquisition and RNA isolation

Tissue samples from oesophageal cancers and corresponding normal tissues were collected by endoscopic biopsy prior to starting neoadjuvant treatment. Samples were snap-frozen in liquid nitrogen and stored at −80°C until further processing. Samples were carefully chosen after control staining with haematoxylin and eosin of individual biopsies, and contained >50% tumour cells.

Total cellular RNA was isolated using Trizol reagent (Life Technologies/GIBCO, Grand Island, NY, USA) and quantitated at A_260/280 nm_ (Smart Spec; Biorad, Hercules, CA, USA).

### Real-time RT–PCR assay

Total cellular RNA (0.5 *μ*g) was reverse-transcribed using an oligo (dT)_18_ primer and Moloney murine leukaemia virus (MMLV) reverse transcriptase (Clontech Lab, Palo Alto, USA), according to the manufacturer's recommendation. Placenta RNA from this kit was used to prepare standard curves. An amount of 25 ng of cDNA was taken for real-time PCR using the Light Cycler System (Roche, Mannheim, Germany). Amplification was monitored by SybrGreen intercalation. For hot start LC-DNA, Master SYBR Green was preincubated with TaqStart™ antibody (Clontech Lab, Palo Alto, USA), as suggested by the manufacturer. In addition, the 10 *μ*l reaction volume contained 2 mM MgCl_2_ and 1 *μ*M of each primer. Primers used for PCR amplification were chosen to encompass intron between exon sequences to identify false-positive DNA amplification.

Three sets of published primers EGFR-F/EGFR-R (Pawlowski *et al*, 2000), ERB-2/ERB-3 (Ninomiya *et al*, 1992) and P5/P6 (Tokuchi *et al*, 1999) were purchased (Eurogentec, Seraing, Belgium) and used to amplify EGFR mRNA, c-erbB-2 mRNA and *β*-actin mRNA, respectively. A 250 bp amplification product was obtained for c-erbB-1 mRNA, a 183 bp product for c-erbB-2 and a 276 bp product for *β*-actin mRNA. We used the expression of *β*-actin mRNA to normalise the level of expression of EGFR and c-erbB-2 mRNA. Thermal cycling conditions for EGFR were 30 s at 95°C for initial denaturation, followed by 35 cycles of 95°C for 0 s, 68°C for 20 s and 72°C for 20 s; for c-erbB-2, 30 s at 95°C for initial denaturation, followed by 35 cycles of 95°C for 0 s, 64°C for 20 s and 72°C for 20 s; and for *β*-actin, 30 s at 95°C, followed by 40 cycles of 95°C for 0 s, 63°C for 20 s and 72°C for 20 s. We used serial dilutions of standard cDNA synthesised from human placenta total cellular RNA (Clontech Lab Inc, Palo Alto, CA, USA) for the quantification analysis. Triplicates of the clinical samples and duplicates of standard cDNA dilutions were assayed in each run. Product purity was controlled by melting point analysis and PCR products were further analysed electrophoretically in 2% agarose gels using a Horizon 58 electrophoresis chamber (GIBCO/BRL, Eggenstein, Germany) and visualised by ethidium bromide staining.

Absolute mRNA expression levels were calculated as c-erbB-1 or c-erbB-2/*β*-actin in tumour and normal tissue, respectively, and relative mRNA expression levels (ratio tumour/normal) were calculated as (c-erbB-1 or c-erbB-2/*β*-actin in tumour)/(c-erbB-1 or c-erbB-2/*β*-actin in paired normal tissue).

### Statistical analysis

Gene expression levels were described using the median as point estimator and the range of values. Cutoff values for discrimination of mRNA expression levels and histopathologic response were derived from receiver operating curve (ROC) data (area under the curve and the 95% confidence interval) according to Metz *et al* (1973).

Associations between dichotomised gene expression levels and clinico-pathological parameters were evaluated using *χ*^2^-analysis and Fisher's exact test for significance (Software Package SPSS for Windows, Version 11.0, Chicago, IL, USA).

*P*-values are given for two-sided testing and the level of significance was set to *P*<0.05.

## RESULTS

### Distribution of c-erbB-1 and c-erbB-2 mRNA expression levels

Quantitative expression levels of c-erbB-1 and c-erbB-2 mRNA were evaluated in 36 patients in tumour and corresponding normal tissues from pretreatment biopsies. The Median absolute c-erbB-1 mRNA expression levels standardised for *β*-actin were 0.115 (min. 0.001, max. 2) in tumour and 0.103 (min. 0.003, max. 1.467) in the corresponding normal tissue. The calculated median relative expression level (*T*/*N* ratio) was 1.082 (min. 0.008, max. 27.148). For c-erbB-2 mRNA expression, median absolute levels standardised for *β*-actin were 0.401 (min. 0.002, max. 7) in tumour and 0.634 (min. 0.11, max. 62.091) in paired normal tissues. The calculated median relative c-erbB-2 mRNA expression level (*T*/*N* ratio) was 0.595 (min. 0.007, max. 7.873).

### Histopathologic response to radiochemotherapy and c-erbB-1 mRNA expression

Relative overexpression of c-erbB-1 mRNA with a *T*/*N* ratio >1.0 was detected in 20 out of 36 (55.6%) tumours and normal to low relative expression with a *T*/*N* ratio ⩽1.0 in 16 out of 36 (44.4%), respectively. The association of dichotomised relative expression levels (*T*/*N* ratio⩽1.0 *vs* >1) and histopathologic response grades is shown in Table 2

**Table 2:** c-erbB-1 mRNA expression and regression (sensitivity and specificity of response prediction)

	**Regression grading**		
**c-erbB-1^a^**	**Grades I/II (MiHR^b^)**	**Grades III/IV (MaHR^c^)**	**Total: *n* (%)**
⩽1: *n* (%)	11 (30.6%)	5 (13.9%)	16 (44.4%)
>1: *n* (%)	13 (36.1%)	7 (19.4%)	20 (55.6%)
Total: *n* (%)	24 (66.7%)	12 (33.3%)	36 (100%)

a Dichotomised c-erbB-1 mRNA levels.

b Minor histopathologic response.

c Major histopathologic response.

Prediction of MiHR (c-erbB1>1): sensitivity 54.1%, specificity: 65% *χ*^2^-analysis (Fisher's exact test): *P*=1.

. This association is statistically not significant (*P*=1), with a low sensitivity (54.1%) and specificity (65%) to predict minor histopathologic response.

A cutoff value for relative c-erbB-1 mRNA expression and discrimination of minor histopathologic response was derived from ROC curves for a relative c-erbB-1 mRNA expression level of 3.318 (area under the curve: 0.476; 95% confidence interval: 0.29–0.66). The results are summarised in Table 3

**Table 3:** c-erbB-1 mRNA expression and regression (sensitivity and specificity of response prediction for maximum cutoff)

	**Regression grading**		
**c-erbB-1^a^**	**Grades I/II (MiHR^b^)**	**Grades III/IV (MaHR^c^)**	**Total: *n* (%)**
⩽3.318: *n* (%)	19 (52.8%)	12 (33.3%)	31 (86.1%)
>3.318: *n* (%)	5 (13.9%)	∅	5 (13.9%)
Total: *n* (%)	24 (66.7%)	12 (33.3%)	36 (100%)

a Dichotomised c-erbB-1 mRNA levels.

b Minor histopathologic response.

c Major histopathologic response.

Prediction of MiHR (c-erbB1>3.32): sensitivity 20.8%, specificity: 100% *χ*^2^-analysis (Fisher's exact test): *P*=0.15.

and show no statistically significant association between dichotomised expression levels (*T*/*N* ratio⩽3.318 *vs* >3.318). The sensitivity to predict minor histopathologic response was 20.8%, with a specificity of 100% (*P*=0.15).

There was no significant association between dichotomised mRNA levels for *T*/*N* ratios ⩽1.0 *vs* >1 and histologic type (*P*=0.17), pT categories (*P*=1), pN categories (*P*=0.25) and grading (*P*=1) and *T*/*N* ratios ⩽3.32 *vs* >3.32 and histologic type (*P*=0.63), pT categories (*P*=0.0.63), pN categories (*P*=1) and grading (*P*=0.65) by Fisher's exact test.

### Histopathologic response to radiochemotherapy and c-erbB-2 mRNA expression

The response frequencies for the 36 tumours analysed for c-erbB-2 expression were as follows: 12 out of 36 (33.3%) of the tumours demonstrated major (grades III and IV) and 24 out of 33 (66.7%) minor histopathologic response (grades I and II) to our neoadjuvant treatment regimen.

Relative overexpression of c-erbB-2 mRNA with a *T*/*N* ratio >1.0 was detected in 10 out of 36 (27.8%) tumours, and normal to low relative expression with a *T*/*N* ratio ⩽1.0 in 26 out of 36 (72.2%), respectively. In addition, a cutoff value for relative c-erbB-2 mRNA expression and maximum discrimination of minor histopathologic response was calculated from ROC curves for a relative c-erbB-2 mRNA expression level of 1.06 (area under the curve: 0.62; 95% confidence interval: 0.42–0.81). This calculated cutoff value, however, showed identical results when compared to dichotomised expression levels at *T*/*N* ratios ⩽1.0 *vs* >1 (Table 4

**Table 4:** c-erbB-2 mRNA expression and regression (sensitivity and specificity of response prediction)

	**Regression grading**		
**c-erbB-2^a^**	**Grades I/II (MiHR^b^)**	**Grades III/IV (MaHR^c^)**	**Total: *n* (%)**
⩽1 or ⩽1.06: *n* (%)^d^	14 (38.9%)	12 (33.3%)	26 (72.2%)
>1 or >1.06: *n* (%)^d^	10 (27.8%)	∅	10 (27.8%)
Total: *n* (%)	24 (66.7%%)	12 (33.3%)	36 (100%)

a Dichotomised c-erbB-2 mRNA levels.

b Minor histopathologic response.

c Major histopathologic response.

d All values remain identical if maximum cutoff at 1.06 was chosen from ROC curves. *χ*^2^-analysis (Fisher's exact test): *P*=0.015.

Prediction of MiHR (c-erbB2 >1): sensitivity 41.6%, specificity: 100%.

).

There was no significant association between dichotomised mRNA levels for *T*/*N* ratios ⩽1.0 *vs* >1 or ⩽1.06 *vs* >1.06 and histologic types (*P*=1.0), pT categories (*P*=0.93), pN categories (*P*=0.21) and grading (*P*=1).

Table 4 shows the association between major and minor histopathologic response groups and dichotomised relative c-erbB-2 expression levels for the whole study group (*n*=36), Table 5

**Table 5:** c-erbB-2 mRNA expression and regression in SCC^a^ (sensitivity and specificity of response prediction)

	**Regression grading**		
**c-erbB-2^b^**	**Grades I/II (MiHR^c^)**	**Grades III/IV (MaHR^d^)**	**Total: *n* (%)**
⩽1 or ⩽1.06^e^: *n* (%)	8 (34.8%)	9 (39.1%)	17 (73.9%)
>1 or >1.06^e^: *n* (%)	6 (26.1%)	∅	6 (26.1%)
Total: *n* (%)	14 (60.9%)	9 (39.1%)	23 (100%)

a Squamous cell cancer.

b Dichotomised c-erbB-2 mRNA levels.

c Minor histopathologic response.

d Major histopathologic response.

e All values remain identical if maximum cutoff at 1.06 was chosen from ROC curves. *χ*^2^-analysis (Fisher's exact test): *P*=0.048.

Prediction of MiHR (c-erbB-2 >1): sensitivity 42.8%, specificity: 100%.

for patients with squamous cell histology (*n*=23) and Table 6

**Table 6:** c-erbB-2 mRNA expression and regression in AC^a^ (sensitivity and specificity of response prediction)

	**Regression grading**		
**c-erbB-2^b^**	**Grades I/II (MiHR^c^)**	**Grades III/IV (MaHR^d^)**	**Total: *n* (%)**
⩽1 or ⩽1.06^e^: *n* (%)	6 (46.2%)	3 (23.1%)	9 (69.2%)
>1 or >1.06^e^: *n* (%)	4 (30.8%)	∅	4 (30.8%)
Total: *n* (%)	10 (76.9%)	3 (23.1%)	13 (100%)

a Adenocarcinoma.

b Dichotomised c-erbB-2 mRNA levels.

c Minor histopathologic response.

d Major histopathologic response.

e All values remain identical if maximum cutoff at 1.06 was chosen from ROC curves. *χ*^2^-analysis (Fisher's exact test): not possible (three cells with expected frequencies <5).

Prediction of MiHR (c-erbB-2 >1): sensitivity 40%, specificity: 100%.

for adenocarcinomas (*n*=13). The sensitivity for detection of a minor histopathologic response was 41.6% for the whole group, 42.8% for squamous cell cancers and 40% for adenocarcinomas with a specificity of 100% in all the three groups. This association of dichotomised c-erbB-2 mRNA levels and histopathologic response was significant for the whole group of tumours (*P*<0.015) and the subgroup of squamous cell cancers (*P*<0.048). Statistical analysis was not possible for adenocarcinomas due to small (*n*=13) patient numbers.

**Figure 1:**
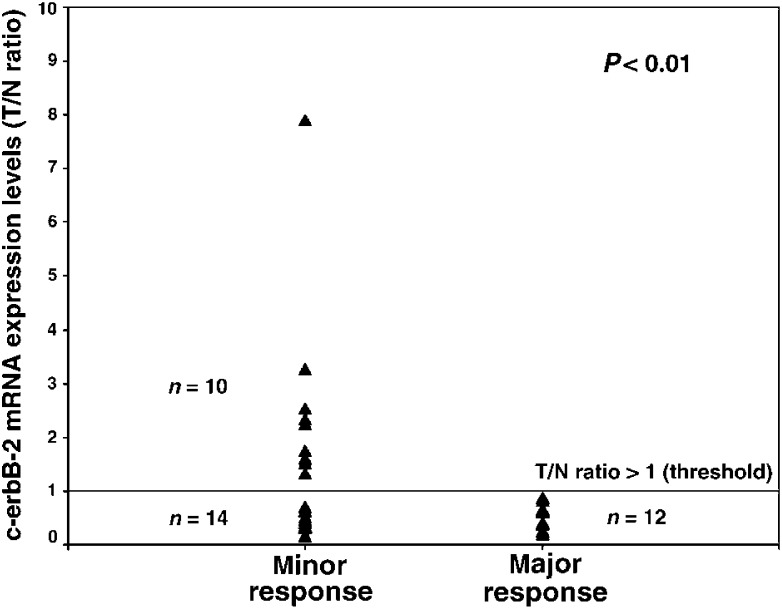
Scattergram showing relative c-erbB-2 mRNA expression levels (*T*/*N*: ratio of tumour to normal tissue) in relation to minor and major histopathologic response in resected specimens. C-erbB-2 expression levels >1 are exclusively present in the group of minor histopathologic response (sensitivity: 41.6%, specificity: 100%).

In summary, quantitative c-erbB2 mRNA expression testing unequivocally identified 10 out of 36 (28%) patients, whose tumours did not respond well to the delivered neoadjuvant radiochemotherapy (Figure 1

).

## DISCUSSION

A significant association between quantitative c-erbB-2 but not c-erbB-1 mRNA expression levels in oesophageal cancers and minor histopathologic response to CDDP/5-FU-based neoadjuvant radiochemotherapy could be demonstrated. In fact, quantitative c-erbB-2 mRNA expression testing by real-time RT–PCR unequivocally identified 10 out of 36 (28%) patients, whose tumours did not exhibit (specificity 100%) a major histopathologic response to the applied preoperative treatment.

Several *in vitro* studies demonstrated that EGF and EGFR expression enhanced the sensitivity to various anticancer agents (Dixit *et al*, 1997; Nouri *et al*, 2000) and radiotherapy (Kwok and Sutherland, 1989; Bonner *et al*, 2002). Kroning *et al* (1995) showed the improvement of drug sensitivity including CDDP and 5-fluorouracil by EGF in various cell lines. Furthermore, they described that enhancement of drug sensitivity depended on the number of receptors. Dixit *et al* (1997) reported that a critical level of EGFR signalling had an inhibitory effect on the repair of CDDP-damaged DNA. Despite positive *in vitro* data, clinical trials failed to demonstrate the relation between EGFR expression and response to chemotherapy with CDDP-containing regimens (Goff *et al*, 1998; Baekelandt *et al*, 1999). There is a possibility for a more complex *in vivo* reaction process between EGFR, cytotoxic agents and radiation-induced DNA damage.

Concerning our negative clinical results, it is necessary to point out that EGFR mRNA expression levels were examined in pretreatment biopsies only, so that we cannot rule out that induction of EGFR expression and signalling during radiochemotherapy as shown *in vitro* could contribute to progressive loss of chemo- and radiation sensitivity during the course of radiochemotherapy. In addition, our data also do not advocate against the potential benefit of an EGFR-targeted therapy (Anderson and Jankowski, 2003; Gee and Nicholson, 2003).

Nevertheless, EGFR mRNA expression testing in pretreatment biopsies did not significantly contribute to the identification of patients with major or minor response to our radiochemotherapy protocol.

Contrary to the c-erbB-1 expression data, increased relative c-erbB-2 mRNA levels prior to the beginning of preoperative radiochemotherapy proved to be a statistically significant factor in predicting minor histopathologic response (*P*<0.01) in our clinical setting.

It is known from previous studies that c-erbB-2 overexpression can induce chemoresistance against various cytotoxic agents (Allred *et al*, 1992; Benz *et al*, 1993; Miles *et al*, 1999; Menard *et al*, 2001) and several groups demonstrated that anti-c-erbB2 (p185)-specific antibodies enhanced the cytotoxic sensitivity in high p185 protein-expressing cell lines (Hancock *et al*, 1991; Pietras *et al*, 1994; Tsai *et al*, 1995). Tsai *et al* (1996) concluded from *in vitro* experiments using non-small cell lung cancer cell lines that high levels of p185 can promote DNA repair after exposure to cytotoxic agents.

In clinical studies, it has been shown that overexpression of c-erbB-2 protein induced chemoresistance in breast and ovarian cancers (Muss *et al*, 1994; Meden *et al*, 1998; Jukkola *et al*, 2001). Other studies, however, failed to demonstrate a relation between c-erbB-2 expression and chemotherapy response in breast, ovarian or squamous cell head and neck cancers (Makar *et al*, 1994; Rozan *et al*, 1998; Giatromanolaki *et al*, 2000).

Clinical response evaluation after neoadjuvant therapy in solid tumours is highly inaccurate, as shown for oesophageal cancer (Adelstein *et al*, 1997). In contrast, histopathologic evaluation results in an objective analysis of remission with prognostic importance, as convincingly demonstrated for non-small cell lung cancer (Junker *et al*, 1997; Thomas *et al*, 1999). PET imaging might further improve clinical response evaluation, which, however, is currently still under investigation (Bruecher *et al*, 2001; Downey *et al*, 2003).

We therefore applied histopathologic criteria instead of inaccurate clinical restaging modalities for objective response evaluation in neoadjuvant-treated oesophageal cancers and identified a significant association between minor histopathologic response and dichotomised relative c-erbB-2 mRNA expression levels (*P*<0.01).

The sensitivity for the whole group of oesophageal cancers is 41.6%. More important, however, is the specificity of 100%, which would allow the unequivocal identification of a subset of 28% of patients with minor responses prior to treatment. Although median follow-up is too short to allow a definitive evaluation between the association of c-erbB-2 expression levels and survival, it has been convincingly demonstrated in the past that only patients with major histopathologic responses benefit from this type of treatment independent of the applied protocol (Walsh *et al*, 1996; Urba *et al*, 2001; MRC Esophageal Study Group, 2002).

The present, still early, results of this ongoing study are promising, and it appears that we could expect to identify approximately one-fourth of patients who in principal fulfil the criteria for neoadjuvant treatment for locally advanced oesophageal cancer, who will, however, not benefit from our treatment protocol. This might prevent a substantial number of our patients from expensive, noneffective and potentially harmful therapies, and could lead to a more individualised type of combined multimodality treatment in the near future. Quantitative real-time RT–PCR is a very efficient and fast technology that could easily be incorporated in a clinical setting, since results can be obtained within 1 or 2 days. A very promising approach could also be the application of specific anti-p185 antibodies or tyrosine kinase inhibitors in addition to radiochemotherapy in patients overexpressing c-erbB-2.
